# Prótese Mecânica X Prótese Biológica: Uma Decisão Individualizada e Compartilhada

**DOI:** 10.36660/abc.20210488

**Published:** 2021-07-15

**Authors:** João Ricardo C. Fernandes, Roney Orismar Sampaio

**Affiliations:** 1 Universidade de São PauloFaculdade de MedicinaHospital das ClinicasSão PauloSPBrasilUniversidade de São Paulo - Faculdade de MedicinaHospital das Clinicas Instituto do Coração, São Paulo, SP - Brasil

**Keywords:** Bioprótese, Implante de Prótese de Valva Cardíaca/complicações, Próteses Valvulares Cardíacas, Epidemiologia, Valva Aórtica, Febre Reumática

As intervenções cirúrgicas valvares são realizadas principalmente em pacientes com valvopatia grave, com sintomas associados e/ou efeitos anatômicos/hemodinâmicos, sem contraindicação ao procedimento. A escolha da válvula protética deve ser baseada em um processo de tomada de decisão compartilhado que deve levar em conta as compensações entre durabilidade (e a necessidade de reintervenção), sangramento e tromboembolismo.

O manuscrito: “Evolução tardia das próteses biológicas e mecânicas em posição aórtica”^1^ é um estudo observacional retrospectivo que visa avaliar o seguimento a longo prazo de pacientes (n = 202) submetidos à troca valvar aórtica, comparando as próteses biológicas (65,3%) e mecânicas (34,7%). Os pacientes eram relativamente jovens (média de idade: 49 anos), predominantemente com doença valvar aórtica degenerativa. Por outro lado, 22% da população estudada apresentava doença reumática, a etiologia mais prevalente de valvopatia no Brasil.^2^

Os autores não encontraram nenhuma diferença nas taxas de mortalidade após 10 anos de seguimento; entretanto, o implante de prótese mecânica foi associado a uma menor taxa de reoperação (sem reoperação no grupo da prótese mecânica). Ao se analisar especificamente as reoperações de biopróteses, os pacientes com menos de 30 anos apresentaram as maiores taxas, comparados aos maiores de 50 anos, e até mesmo aos pacientes entre 30 e 49 anos. Uma meta-análise recente descobriu que a idade mais jovem era um fator de risco significativo para degeneração estrutural da válvula aórtica, além da área de superfície corporal, tabagismo e incompatibilidade paciente-prótese.^3^ No presente estudo, 50% do subgrupo de pacientes com menos de 30 anos foi submetido a reoperação em 10 anos.

Na mesma direção, estudos baseados em seguimentos por ecocardiogramas transtorácicos estimam que aproximadamente 30% dos pacientes com implante cirúrgico de bioprótese valvar aórtica desenvolvem evidências de disfunção valvar mais de 10 anos após o implante, sendo a idade jovem (<60 anos) um dos fatores de risco associados à deterioração valvar acelerada (<5 anos).^4^ As diretrizes do *American College of Cardiology*/*American Heart Association *para o tratamento de valvopatia recomendam uma avaliação por ecocardiograma transtorácico 5 e 10 anos após o implante cirúrgico de uma válvula bioprotética, e um avaliação mais precoce se houver sintomas ou sinais clínicos que sugiram disfunção da válvula protética.^5^ Entretanto, os pacientes geralmente permanecem assintomáticos até que a disfunção da válvula seja grave o suficiente para resultar em consequências hemodinâmicas adversas ou fibrilação atrial. Porém, no presente estudo, não ficou claro se essa foi a abordagem utilizada, o que poderia ter retardado o diagnóstico de disfunção de prótese.

O risco de sangramento foi maior no grupo da prótese mecânica (20,97% x 5,38%), enquanto não houve diferença nas taxas de acidente vascular cerebral, vazamento paravalvar, trombose ou endocardite entre os dois grupos. Curiosamente, não houve trombose no grupo da prótese mecânica, embora dois pacientes não estivessem recebendo terapia de anticoagulação oral. Embora seja uma complicação incomum, a trombose valvar é mais frequentemente observada em pacientes com próteses mecânicas.^6^

Não houve diferenças em relação ao risco de acidente vascular cerebral (AVC) isquêmico entre os grupos. No entanto, as taxas foram relativamente altas (14,1% para próteses biológicas e 11,5% para próteses mecânicas). Seria interessante e relevante saber se esses pacientes tinham fibrilação atrial documentada antes da ocorrência do AVC e também se faziam uso de anticoagulação oral, informação não disponível no estudo.

Em geral, este tópico é muito desafiador e relevante. Apesar da taxa significativamente maior de deterioração estrutural da válvula bioprotética observada em pacientes mais jovens *versus* pacientes mais velhos, muitos pacientes mais jovens optam por evitar receber uma prótese mecânica, seja porque não desejam considerar a terapia com varfarina em longo prazo ou devido às inconveniências de restrições relacionadas a monitoramento, gravidez, alimentares, interações medicamentosas e a necessidade de restringir a participação em alguns tipos de atividades atléticas. Como mencionado anteriormente, a escolha da válvula protética deve ser baseada em um processo de tomada de decisão compartilhado que leva em consideração os valores e preferências do paciente. Mais recentemente, a disponibilidade de procedimentos transcateter, com a crescente experiência com o implante *valve-in-valve* na válvula aórtica, adiciona outra questão a ser levada em consideração na decisão do tipo de prótese valvar. A substituição transcateter *valve-in-valve* da válvula aórtica surgiu como uma alternativa menos invasiva para refazer a substituição cirúrgica em pacientes inoperáveis ou de alto risco com bioprótese degenerativa. Um estudo recente mostrou que cerca de 3 em cada 4 pacientes sobreviveram após um seguimento médio de 3 anos, com a hemodinâmica da válvula permanecendo estável ao longo do tempo, e houve uma baixa taxa de doença valvar estrutural clinicamente relevante, apoiando o procedimento transcateter *valve-in-valve* da válvula aórtica como uma alternativa para refazer a substituição cirúrgica para o tratamento de falha da válvula aórtica bioprotética.^7^

Em última análise, uma limitação importante do presente estudo é a falta de informações sobre o tempo na faixa terapêutica para pacientes em uso de anticoagulação. O nível socioeconômico tem grande influência na adesão ao tratamento. Um estudo brasileiro mostrou que apenas cerca de um terço dos pacientes apresentava nível adequado de anticoagulação em mais da metade das consultas.^8^

Devido ao seu desenho retrospectivo, não-randomizado, unicêntrico e observacional, o presente estudo é insuficiente para permitir conclusões robustas. Mesmo assim, ele aborda uma etapa importante da tomada de decisão no manejo da doença cardíaca valvar.
