# Supplementary material for: Prótese Mecânica X Prótese Biológica: Uma Decisão Individualizada e Compartilhada
Source: Arq Bras Cardiol. 2021 Jul 15;117(1):37–8. [Article in Portuguese] doi: 10.36660/abc.20210488 (PMC8294719; doi:10.36660/abc.20210488)
Supplement: Translation

# Translation: Mechanical Prosthesis X Biological Prosthesis: an Individualized and Shared Decision

**Authors:** João Ricardo C. Fernandes, Roney Orismar Sampaio

**Affiliations:** 1 BrasilUniversidade de São Paulo - Faculdade de Medicina Hospital das Clinicas Instituto do Coração, São Paulo, SP - Brasil

**Keywords:** Bioprosthesis, Heart Valve Prosthesis Implantation/complications, Heart Valve Prosthesis, Epidemiology, Aortic Valve, Rheumatic Fever

Surgical valve interventions are performed primarily in patients with severe valve heart disease, with associated symptoms and/or anatomical/hemodynamic consequences, and no contraindication to the procedure. The choice of prosthetic valve should be based on a shared decision-making process that must account for the trade-offs between durability (and the need for reintervention), bleeding, and thromboembolism.

The manuscript: “*Late Outcomes of Aortic Valve Replacement with Bioprosthesis and Mechanical Prosthesis*”^1^ is an observational retrospective study intended to evaluate the long-term follow-up of patients (n=202) who underwent aortic valve replacement, comparing biological (65.3%) and mechanical prostheses (34.7%). The patients were relatively young (mean age: 49 years), with predominantly degenerative aortic valve disease. On the other hand, 22% of the study population had rheumatic disease, the most prevalent etiology of valve disease in Brazil.^2^

The authors found no difference in mortality rates after 10 years of follow-up; however, the implantation of a mechanical prosthesis was associated with a lower rate of reoperation (with no reoperation in the mechanical prosthesis group). When analyzing specifically the bioprosthesis reoperations, patients under 30 years old had the highest rates, compared to those older than 50 years, and even to patients between 30 and 49 years of age. A recent meta-analysis found that younger age was a significant risk factor for aortic structural valve degeneration, besides body surface area, smoking and patient-prosthesis mismatch.^3^ In the present study, 50% of the subgroup of patients under 30 years old was reoperated in 10 years.

In the same direction, studies based on transthoracic echocardiogram follow-up estimate that approximately 30% of patients with a surgical aortic valve bioprosthesis develop evidence of valve dysfunction over 10 years after implantation, with young age (<60 years) being one of the risk factors associated with accelerated (<5 years) valve deterioration.^4^ The American College of Cardiology / American Heart Association Guideline for the management of valvular heart disease recommends a transthoracic echocardiogram evaluation 5 and 10 years after the implantation of a bioprosthetic surgical valve, and an earlier evaluation if there are clinical symptoms or signs that suggest prosthetic valve dysfunction.^5^ However, patients typically remain asymptomatic until valve dysfunction is severe enough to result in adverse hemodynamic consequences or atrial fibrillation. However, in the present study, it was not clear whether this was the approach used, which could have delayed the diagnosis of a prosthesis dysfunction.

Bleeding risk was higher in the mechanical prosthesis group (20.97% x 5.38%), while there was no difference in the rates of stroke, paravalvular leak, thrombosis or endocarditis between the two groups. Interestingly, there was no thrombosis in the mechanical prosthesis group, even though two patients were not on oral anticoagulation therapy. Although it is an uncommon complication, valve thrombosis is more frequently observed in patients with mechanical prosthesis.^6^

There was no difference regarding the risk of ischemic stroke between the groups. However, the rates were relatively high (14.1% for bioprosthesis and 11.5% for mechanical prosthesis). It would be interesting and relevant to know whether those patients had documented atrial fibrillation before the occurrence of stroke and also if they were on oral anticoagulation therapy, an information not available in the study.

In general, this topic is very challenging and relevant. Despite the significantly higher rate of bioprosthetic structural valve deterioration observed in younger *versus* older patients, many younger patients choose to avoid receiving a mechanical prosthesis, either because they are unwilling to consider long-term warfarin therapy or due to the inconvenience of monitoring, pregnancy, dietary restrictions, medication interactions, and the need to restrict participation in some types of athletic activities. As mentioned before, the choice of prosthetic valve should be based on a shared decision-making process that accounts for the patient’s values and preferences. More recently, the availability of transcatheter procedures, with the growing experience with aortic valve-in-valve procedure, adds another issue to be taken into account when deciding the type of valve prosthesis. Transcatheter valve-in-valve aortic valve replacement has emerged as a less invasive alternative to redoing surgical replacement for inoperable or high-risk patients with degenerative bioprosthesis. A recent study showed that about 3 out of 4 patients survived after a median follow-up of 3 years, with valve hemodynamics remaining stable over time, and there was a low rate of clinically relevant structural valve disease, supporting the transcatheter aortic valve-in-valve procedure as an alternative to redoing surgical replacement for treating bioprosthetic aortic valve failure.^7^

Ultimately, one important limitation of the present study is the lack of information about time in therapeutic range for patients receiving anticoagulation. The socioeconomic status has a great influence on treatment adherence. A Brazilian study showed that only approximately one-third of the patients showed an adequate anticoagulation level in more than half of the consultations.^8^

Because of its retrospective, nonrandomized, unicentric and observational design, the present study is insufficient to allow drawing robust conclusions. Even though, it addresses an important step of decision-making in valvular heart disease management.
